# Left atrial intramural hematoma after percutaneous coronary intervention

**DOI:** 10.1002/ccr3.4654

**Published:** 2021-08-15

**Authors:** Jun Takaki, Takashi Yoshinaga, Kosaku Nishigawa, Ken Okamoto, Toshihiro Fukui

**Affiliations:** ^1^ Department of Cardiovascular Surgery Kumamoto University Hospital Kumamoto Japan

**Keywords:** complications, PCI, surgery

## Abstract

Left atrial intramural hematoma is a rare complication of percutaneous coronary intervention. The combination treatment with surgery and interventional therapy is one of therapeutic options.

## Graphical abstract

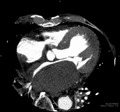

## 1 INTRODUCTION

Left atrial intramural hematoma is a rare complication of percutaneous coronary intervention. We report a case of left atrial intramural hematoma after percutaneous coronary intervention that was successfully treated by combining surgery with interventional therapy.

Left atrial intramural hematoma (LA IMH) as a complication of percutaneous coronary intervention (PCI) is a very rare entity. We, herein, report a case of LA IMH after PCI that was successfully treated by combining surgery with interventional therapy.

## 2 CASE REPORT

A 79‐year‐old man with a history of hypertension, diabetes, chronic kidney disease, aplastic anemia, and thrombocytopenic purpura was admitted to another hospital with severe chest pain. He was diagnosed with unstable angina and underwent immediate coronary angiography. There was a 75% stenosis in the right coronary artery (RCA), and 90% stenosis in the left anterior descending artery (LAD) and left circumflex artery (LCX). PCI for LAD and LCX was successfully performed.

**FIGURE 1:**
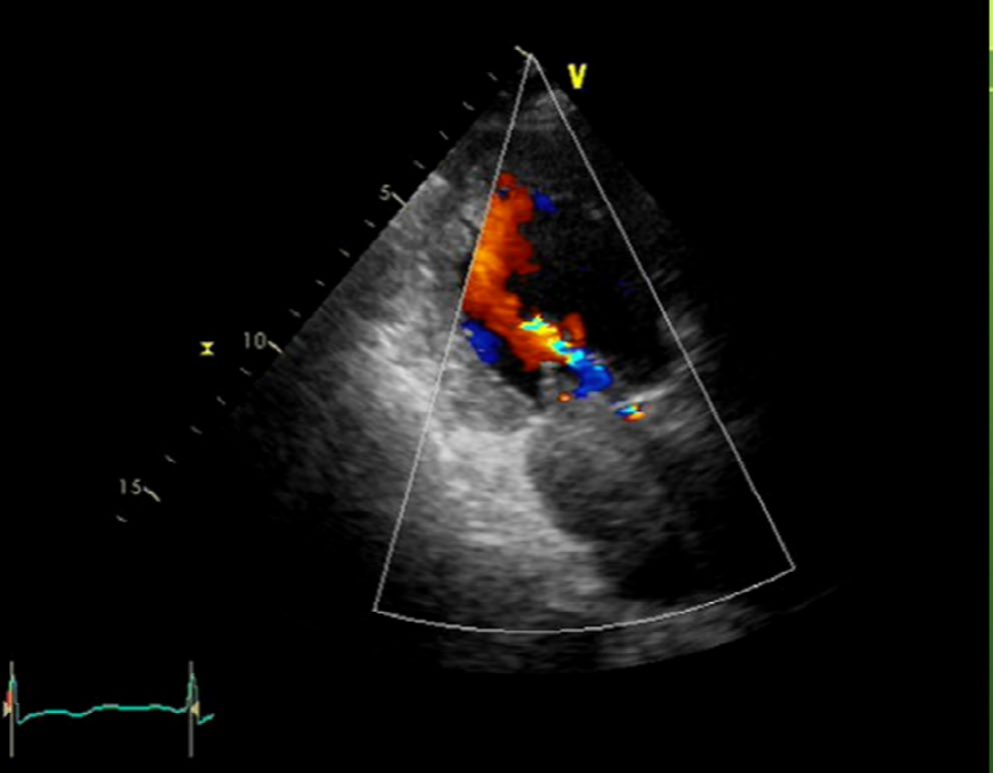
Transthoracic echocardiogram showing an intramural hematoma occupying left atrium preoperatively

Ten hours after the PCI, the patient complained of severe dyspnea. Echocardiogram revealed that a mass occupied the LA cavity (Figure 1). The patient was transferred to our hospital for the treatment of left atrial mass causing acute heart failure.

**FIGURE 2:**
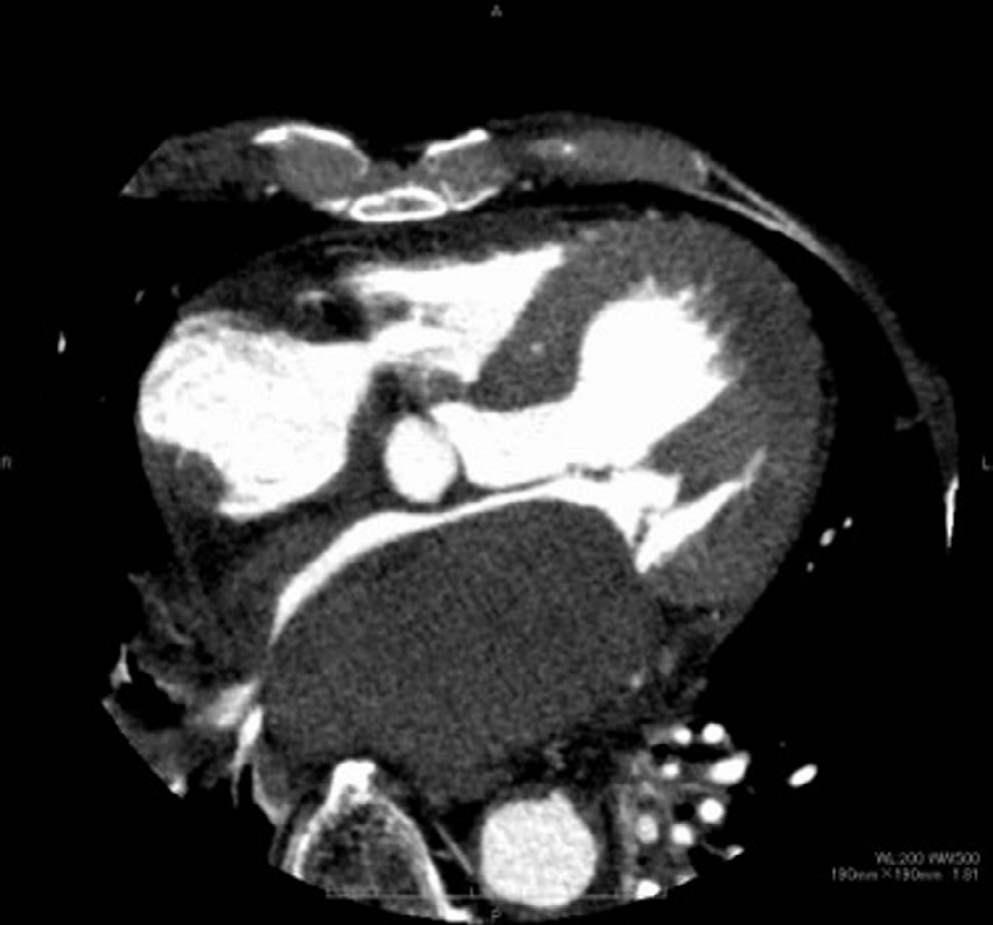
Preoperative computed tomography showing a large left atrial mass

At presentation, he was hemodynamically stable on dobutamine of 1γ and oxygen (blood pressure 100/60 mmHg, pulse 90 beats/min, and saturation 95%). Echocardiography showed that the mean pressure gradient through the mitral valve was 8.3 mmHg and estimated systolic pulmonary arterial pressure was 50 mmHg. Computed tomography (CT) showed a large mass (88 × 60 mm) in the LA (Figure 2); however, no contrast effect was seen in the mass. The patient was diagnosed with acute heart failure, due to the LA mass causing functional mitral stenosis (MS), and underwent emergent surgery for mass removal.

Under median sternotomy, cardiopulmonary bypass was established with ascending aortic and bicaval cannulations. After aortic clamping, the incision of LA was made through the superior transseptal approach. Inside the LA, tumor was not detected and the endocardium was intact. However, a remarkable bulging of the entire posterior wall of the LA was observed and the endocardium of the LA wall was incised. There was a voluminous clot in the LA wall. After complete evacuation of the clot, the endocardium of the posterior wall was sewn with 5–0 polypropylene. Additionally, we performed coronary artery bypass grafting (CABG) for RCA using a saphenous vein graft. Cardiopulmonary bypass and operation time was 160 and 279 min, respectively.

**FIGURE 3:**
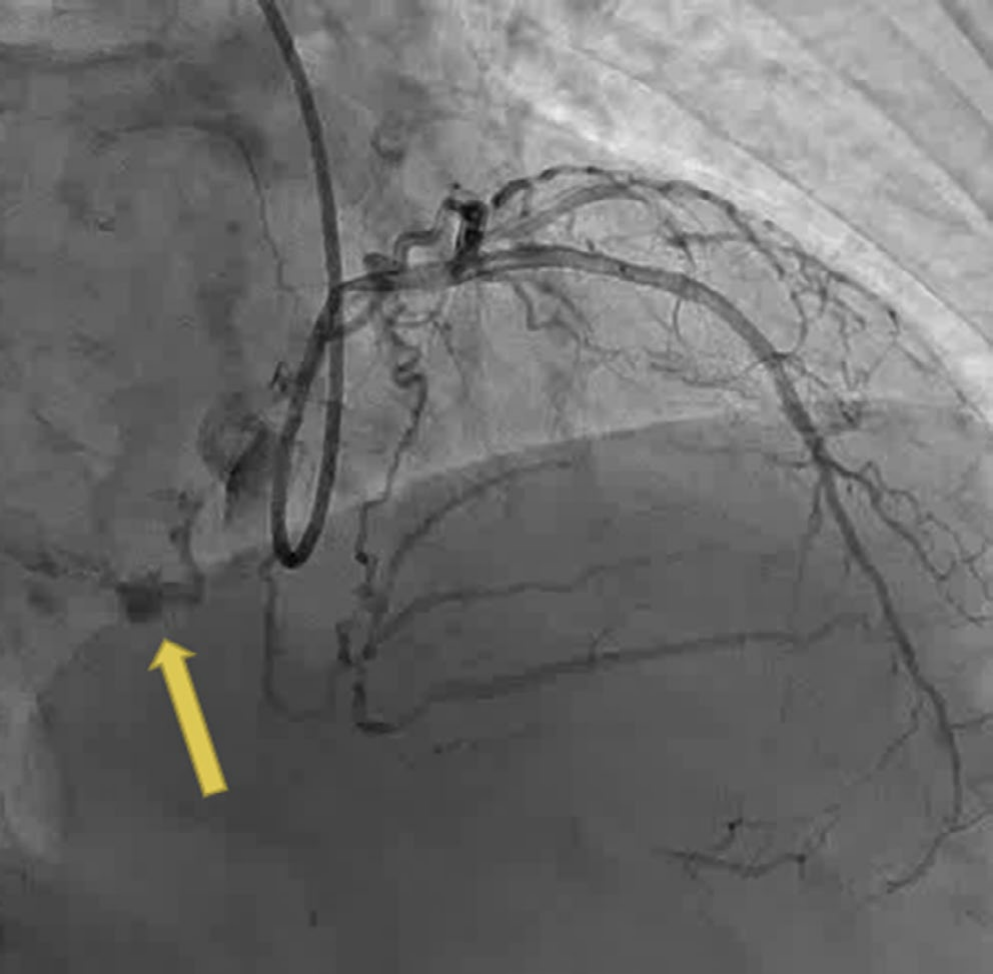
Angiography showing perforation of the terminal left circumflex artery (arrow)

After the operation, we performed angiography again to detect the cause of LA IMH. The angiography showed continuous extravasation of contrast from the terminal LCX (Figure 3), and it was successfully treated with embolization using two 15 mm coils.

**FIGURE 4:**
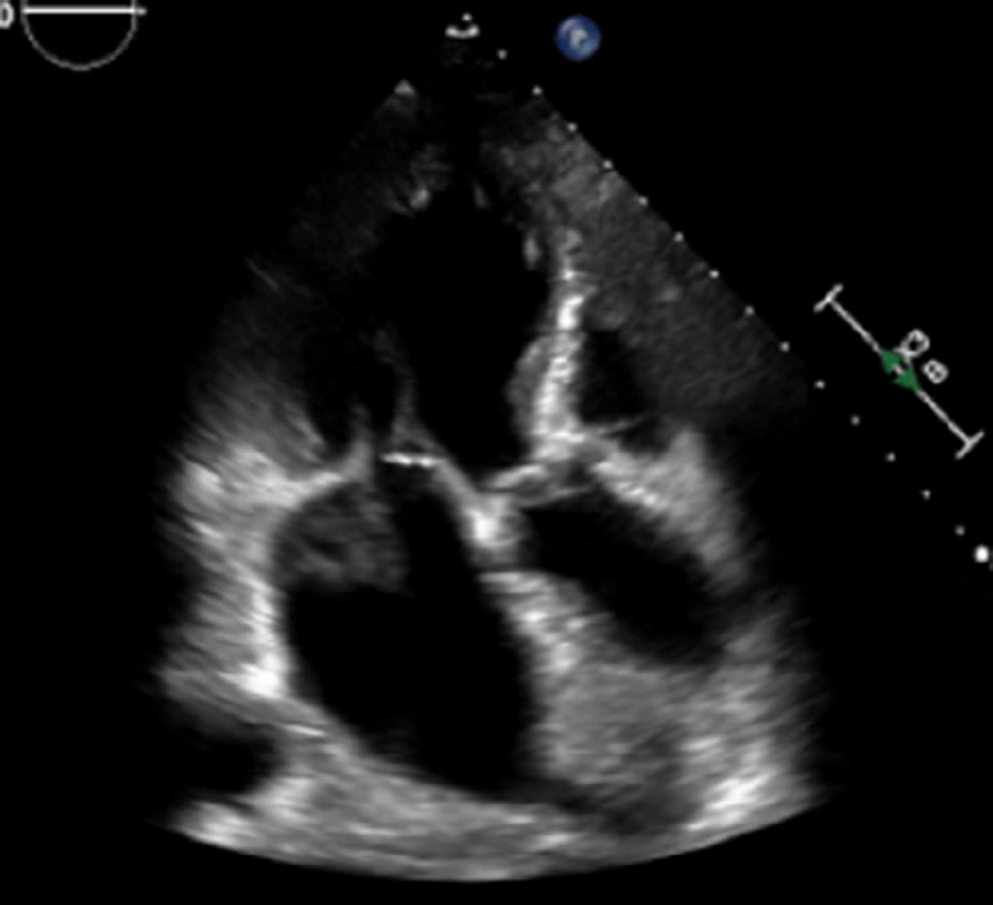
Transthoracic echocardiogram showing the improvement in hematoma postoperatively

The day after surgery, echocardiography showed the LA IMH (42 × 48 mm), however, there was no pressure gradient through the mitral valve. In addition, he remained hemodynamically stable. Because he had to receive dual antiplatelet therapy (aspirin and clopidogrel) for the stent in the coronary artery, we decided to pursue a watchful follow‐up during hospitalization. Serial echocardiography showed improving LA IMH (Figure 4). He was discharged on postoperative day 15.

**FIGURE 5:**
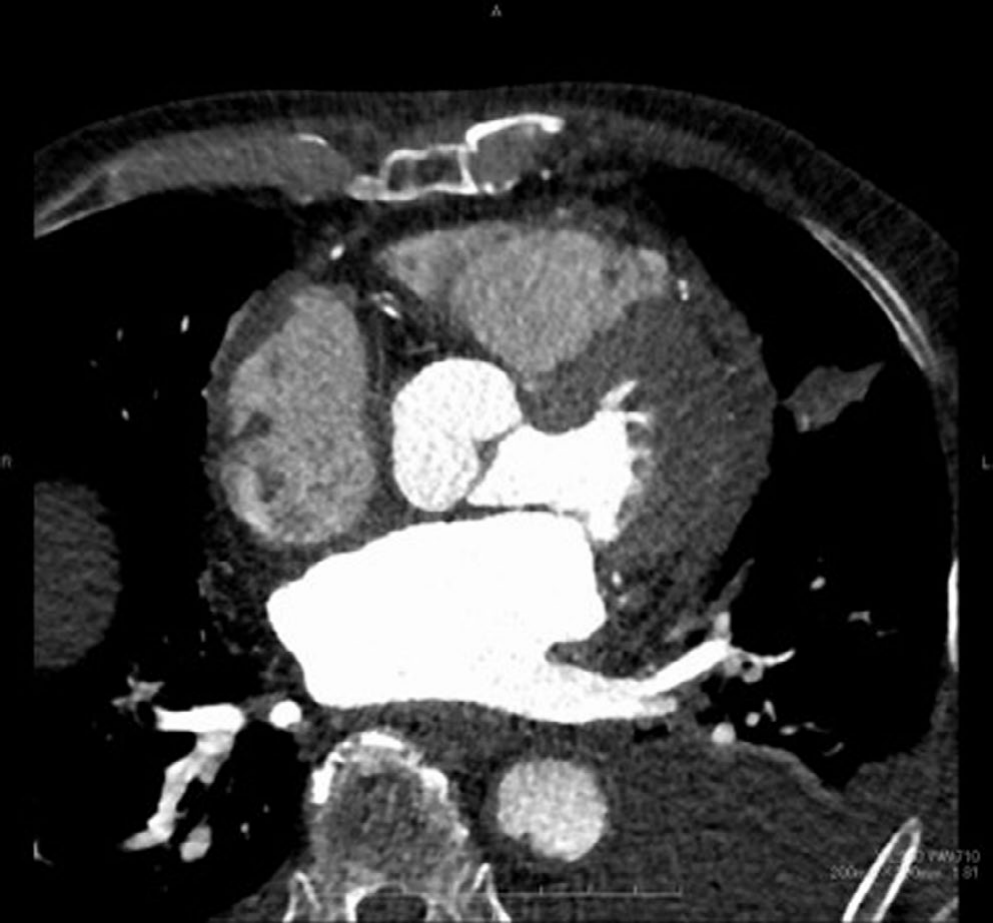
Follow‐up computed tomography showing no mass in the left atrium

One year after the surgery, CT showed no IMH in the LA (Figure 5). Informed consent was obtained from the patient regarding the publication of this case report.

## 3 DISCUSSION

Intramural hematoma is an uncommon cause of LA mass.^1^ There have been several reports of LA IMH occurring as a complication of PCI and ablation.^1^, ^2^, ^3^, ^4^, ^5^

Because the clinical course and outcome of LA IMH are poorly understood, no definitive criteria exist to guide the management of this rare entity. In case of hemodynamic stability, conservative management should be selected^3^; otherwise, surgical treatment should be chosen if patient is hemodynamically unstable. Hemodynamic instability is mainly caused by functional MS.

Jothidasan et al^6^ reported a case of LA IMH that was successfully treated by surgically removing the clot. In that case, the patient had undergone CABG 19 years ago; therefore, an anterolateral thoracotomy approach with small incision of the LA was selected. Cresce et al^7^ reported good results of surgical treatment of LA IMH with mitral annular detachment, which was caused by the dissection of the atrial wall. In these case reports, no additional treatment for coronary artery perforation was performed after evacuation of the hematoma. We performed angiography after surgery to detect the cause of LA IMH, and found continuous extravasation from the LCX. We believe that a combination of surgery and interventional therapy should be considered to prevent the recurrence of LA IMH. Moreover, long‐term follow‐up should be performed, even if early postoperative hemodynamic status is stable.

## 4 CONCLUSION

Left atrial intramural hematoma is a rare complication of percutaneous coronary intervention. The combination treatment with surgery and interventional therapy is one of therapeutic options in patients with left atrial intramural hematoma after percutaneous coronary intervention.

## CONFLICT OF INTEREST

None declared.

## AUTHOR CONTRIBUTIONS

Jun Takaki, Kosaku NIshigawa, Takashi Yoshinaga, Ken Okamoto involved in drafting article. Toshihiro Fukui involved in critical revision.

## ETHICAL APPROVAL

Written informed consent was obtained from the patient.

## Data Availability

The data that support this case report are available from the author upon reasonable request.
